# Ambiguous definitions of industrial mobile robots: a survey of German-speaking experts

**DOI:** 10.3389/frobt.2026.1837981

**Published:** 2026-08-28

**Authors:** Sven Franke, Jan-Bernd Igelmann, Jérôme Rutinowski, Markus Pauly

**Affiliations:** 1 Department of Mechanical Engineering, TU Dortmund University, Dortmund, Germany; 2 Department of Statistics, TU Dortmund University, Dortmund, Germany

**Keywords:** AGV (automated guided vehicle), AMR (autonomous mobile robot), definitions, mobile robot (MR), survey

## Abstract

Industrial mobile robots are increasingly deployed in warehouses and production sites and interaction takes place with different stakeholders from different disciplines. In such sociotechnical settings, terminology shapes mental models and perceptions that influence trust, interaction, and safe collaboration. Key labels such as driverless transport vehicle (DTV), automated guided vehicle (AGV), autonomous mobile robot (AMR) and mobile robot (MR) are used inconsistently across standards, industry practice and academic literature. It remains unclear how experts actually interpret these terms in professional communication, since the terms are only in part defined by standards and perceived differently throughout the industry. We address this gap with an online survey of 53 mobile robotics experts. Spontaneous associations, the perceived distinctiveness between pairs of terms, consistency with general terminology statements and attributed technical characteristics are identified. The results are contrasted with normative references and interoperability initiatives. The findings indicate systematic, term-specific perceptions. Participants report divergent perceptions, conflicting definitions and a lack of uniform terminology. Experts rated AGV and DTV as largely indistinguishable (relative effect = −0.51/-1), but considered AGV and AMR clearly more separable (0.37/1), while MR functioned as a broad umbrella term that evoked capability-related associations yet remained ambiguous. Based on these ambiguities, we synthesize recommendations for how terminology should be communicated and qualified in industrial mobile robotics to reduce expectation gaps across stakeholder groups and facilitate a joint, uniform understanding.

## 1 Introduction

The discipline of robotics is distinguished into industrial robotics, professional service robotics and personal service robotics (Thrun, 2004). In industrial environments the percentage of industrial robotics that are mobile has increased significantly over the past years, extending their role from isolated automation solutions to systems that operate in close proximity to humans (International Federation of Robotics, 2025). Consequently, discussions and communication about robots now occur not only in academia and at the operational level on the shop floor, but also during system planning, deployment, supervision and management. These discussions have become a central element of effective human–robot interaction (HRI). Prior work in HRI and human factors shows that human interaction with complex technical systems is strongly influenced by user expectations, which shape perceptions of system capabilities (Goodrich and Schultz, 2008). Consequently, imprecise or inconsistent communication about robotic systems can lead to mismatched expectations, reduced trust and potentially unsafe interaction behavior. A key component of this communication is the terminology used to describe industrial mobile robotics. Terms such as driverless transport vehicle (DTV), automated guided vehicle (AGV), autonomous mobile robot (AMR) and mobile robot (MR) are widely used in research, industry and standardization contexts (e.g., International Organization for Standardization, 2021; Verein Deutscher Ingenieure, 2005; Fragapane et al., 2021). The industrial robotics market encompasses the use of robots and automation technologies in manufacturing processes, assembly lines, and other industrial environments (Statista, 2026). While these terms are often treated as technical labels, they implicitly convey assumptions about technical specifications, autonomy, adaptability, navigation capabilities, and interaction potential. As a result, terminology does not merely describe a system’s formal properties but also contributes to the formation of mental models that guide how humans interpret and interact with robotic systems. From a normative perspective, definitions of industrial mobile robotics categories are provided by international and national standardization bodies, such as International Organization for Standardization (ISO) or European Standards (EN), for instance (International Federation of Robotics, 2026). These standards aim to establish clear technical distinctions and ensure interoperability and safety. While standards define what a robot is allowed to do, terminology shapes what humans expect a robot to do. However, these standards represent a top-down, formalized view of robotic systems, whereas mental models emerge bottom-up through experience, communication, and contextual use (Norman, 2014). It therefore remains unclear to what extent formal definitions are shared, recognized, or applied by experts in practice and whether different stakeholder groups associate the same expectations with commonly used terms. Despite the relevance of terminology for HRI, there is a lack of empirical evidence on how key terms in industrial mobile robotics are perceived and interpreted within the expert community. Our work addresses this research gap by investigating the perception of the terms DTV, AGV, AMR and MR among experts in robotics.

The goal of this work is to empirically investigate how experts in mobile robotics perceive and interpret the terms DTV, AGV, AMR and MR. In addition, we aim to identify the expectations regarding technical specifications such as autonomy, adaptability, navigation capabilities and interaction potential associated with each term. We focus on skilled workers, managers and researchers from both industry and academia. These groups actively shape the discourse surrounding industrial mobile robotics through system design, deployment decisions, research publications and standardization activities. By eliciting their perceptions we aim to capture the implicit meanings associated with each term beyond formal definitions.

The contributions of this work are threefold.

1. We present empirical results from a survey of 53 experts in industrial mobile robotics, indicating differences and commonalities in the perception of DTV, AGV, AMR and MR across stakeholder groups.
2. We analyze how these perceptions relate to expectations about the terms and compare the responses with the definitions of standards, thereby highlighting the ambiguities between perception and official definitions.
3. We derive implications for the design, communication, and use of terminology in mobile robotics and propose directions for future research on terminology-driven effects in HRI.

The remainder of this paper is structured as follows. Section 2 reviews related work on mental models and HRI, as well as prior research and standardization efforts concerning industrial mobile robotics. Section 3 describes the survey method, including survey design, participant demographics, and analysis procedures. The results of the expert survey are presented in Section 4 and discussed in the context of expert expectations, their relation to standards, and implications for stakeholders. Finally, Section 5 concludes the paper and outlines directions for future research.

## 2 Related work

This section first motivates why terminology matters in HRI by linking labels to mental models and expectation formation. The second part reviews what key terms for industrial mobile robotics (MR, AGV, AMR and DTV) are important, how they are defined and referenced across standards, regulations, interoperability initiatives, and academic literature, and highlights where normative definitions diverge from everyday use, thereby motivating our empirical survey.

### 2.1 Terminology as a cue for expectations in HRI

The physical existence and design of an entity create conceptual and mental models, which ideally are meant to overlap. The conceptual model is the original, appropriate representation of the target system, whereas the mental model is the individual’s own interpretation of that system, which is primarily shaped by interaction with it. Depending on the technical background, experience and the information being processed, each user’s mental model can be different (Norman, 2014). The mental model enables people to describe, explain and predict the target system. Describing it allows them to define its purpose and form, explain and predict are the factors that influence how it operates as a system and determine its status, meaning and what the system does (Rouse and Morris, 1986). In both professional and private settings, humans interact with robots in a variety of ways, which has led to the emergence of the field of HRI (Goodrich and Schultz, 2008). This gives rise to both conceptual and mental models in HRI. One challenge in HRI is dealing with users’ culture, fears and other value-related considerations within the interaction between humans and robots (Sheridan, 2016). A recurring consequence of differing interactions of humans and robots is an expectations gap: people may overestimate or underestimate a robot’s autonomy, sensing, navigation capabilities, or safety behavior, which can influence trust, reliance and the interpretation of failures. Trust research in HRI emphasizes that trust is closely tied to perceived competence and predictability and can be undermined when system behavior violates expectations regardless of whether the system is working as designed (Liu et al., 2021; Scheutz et al., 2017; Kluge et al., 2024). Crucially, expectations are not shaped only by direct interaction; they are also shaped by language and labels. Names and terms act as cues that frame what people infer about a technology and are therefore not neutral. The choice of label can measurably shift attitudes and expectations, even when the underlying functionality is held constant. In the context of MR used in industry, widely used terms may therefore function as expectation, e.g., by implying different degrees of autonomy, navigation flexibility, or maturity (Liu et al., 2021).

MR deployments in warehouses and industrial settings illustrate particularly well why terminology matters. These systems operate as sociotechnical infrastructures, their impact extends beyond vehicle navigation to work organization, process design, safety procedures, technology integration and daily coordination between multiple stakeholder groups. Classic large-scale deployments demonstrate that value is realized through the orchestration of many moving robots and tightly coupled processes, where human roles and workflows are reconfigured around the robotic system (Wurman et al., 2008). Consequently, communication about MRs is rarely confined to engineering descriptions. Instead, it spans procurement and capability claims, operational planning, safety assessments, training and performance monitoring. Literature frames these developments as sociotechnical change, in which digital technologies and automation reshape decisions at management and operational levels. In such settings, terminology becomes part of the system. It influences what different actors assume a robot will do, what constraints they anticipate and which responsibilities they attribute to humans versus automation (Winkelhaus and Grosse, 2022). In addition, with the increasingly anthropomorphic design of MRs and emerging humanoids in both the private and industrial sectors, expectations regarding capabilities and trustworthiness will continue to change in the future (Shaghaghi et al., 2025; Cantucci et al., 2025).

### 2.2 Normative terminology in standards and practice

A substantial body of work attempts to standardize industrial mobile robotics technologies through vocabulary, as well as documents on safety and interoperability. The term MR is explicitly defined at the vocabulary level: ISO8373 (International Organization for Standardization, 2021) defines an MR as a *“[…] robot able to travel under its own control” and further notes that it “[…] can have means to be remotely controlled.”* This definition is also used by ISO19649:2017 “Mobile robots–Vocabulary” (International Organization for Standardization, 2017). ISO8373 further defines the terms robot, autonomy and industrial robots using different categories. The term AGV is defined under the entry of the mobile platform, which is described as the assembly of components which enable locomotion and can include a chassis and a manipulator (International Organization for Standardization, 2021). The International Federation of Robotics refers to the ISO definitions on its website (International Federation of Robotics, 2026). These broad definitions deliberately do not prescribe a specific navigation concept, control architecture or application domain. This makes MR suitable as a broad category but also prone to ambiguity when used without further qualifiers.

In contrast, AGVs and AMRs are often only mentioned indirectly in standards, especially in safety standards and not through a single, globally dominant definition of the term. ISO3691-4 (International Organization for Standardization, 2023) frames its scope around driverless industrial trucks and explicitly acknowledges that these systems can also be known as AGV or AMR. The same document (ISO3691-4) also ties this scope to industrial truck terminology by referencing trucks as defined in ISO5053-1, a vocabulary framework. Industrial trucks are defined as *“[…] wheeled vehicles having at least three wheels with a powered or non-powered driving mechanism—except those running on rails—which are designed either to carry, tow, push, lift, stack or tier in racks any kind of load and which are controlled either by an operator or by driverless automation.”* (International Organization for Standardization, 2020) Furthermore, the term industrial truck is not used in other documents related to automated or autonomous vehicles. The document also covers many vehicles that are driven manually.

In addition to ISO, Germany has the Association of German Engineers (VDI, Verein Deutscher Ingenieure) which publishes highly regarded guidelines in both German and English. Guideline 2510 (Verein Deutscher Ingenieure, 2005), e.g., is entitled Automated Guided Vehicle Systems and describes individual AGVs as *“[…] floor-supported, self-propelled means of transport which are controlled automatically and guided by a non-contact guidance system. They are used for materials transport, i.e., for pulling and/or carrying goods to be conveyed, by means of active or passive load handling devices.”* The German translation of AGV is not the literal translation but DTV. However, DTV is also used in English publications, mostly by German authors, both on websites and in technical literature (see Hansen and Gillert (2008), Arvato (2020), BMW Group (2024), Ehrhardt Partner Group, (2026)) with the same meaning as AGV. This helps explain why, in multilingual and international contexts, terminological conflation can arise through translation and usage conventions. Such usage provides a plausible mechanism for the AGV–DTV confusion observed in industry discourse. The German translation DTV is still frequently used and sometimes creates different expectations and perceptions than the apparent equivalent AGV, see Section 4. This does not imply that the term is wrong, rather it highlights that terminology in practice can be anchored in community usage alongside and sometimes independent of formal standard documentation.

For AMR, a historical shift in naming is widely reported in industrial discourse. AMR has increasingly been adopted to emphasize onboard autonomy (e.g., dynamic navigation and obstacle handling) relative to guided connotations commonly associated with AGV (Siegwart et al., 2011). This shift is reflected less by a single ISO vocabulary definition and more by how recent standardization efforts frame AMR as a focal object. For instance, ISO/DIS21423 (International Organization for Standardization, 2026c), which is currently under development, explicitly targets interoperability among industrial AMR systems produced by different vendors and extends this to AMR fleet manager equipment and other enterprise resources that communicate with AMRs. Currently, it is not known whether the document will provide an explicit definition of AMRs. In parallel, the document of the American National Standards Institute ANSI/RIAR15.08-1-2020 (Institute, 2020) addresses safety requirements for industrial MRs and defines AGVs as MRs that follow exact guide paths, whether along a physical or a virtual line, and AMRs as MRs that dynamically generate paths based on the current environment and the most efficient route between the AMRs current location and future destinations. While this content applies to the US, in the European Union, *Regulation (EU) 2023/1230 of the European Parliament and of the Council of 14 June 2023 on machinery and repealing Directive* will become binding in January 2027. This introduces new safety requirements for autonomous mobile machines, such as *“[…] it shall be equipped with devices intended to detect any human, domestic animal or any other obstacle in its vicinity, where those obstacles could give rise to a risk to the health and safety of persons or domestic animals or to the safe operation of the machinery or related product.”* These and the other requirements may once again change perceptions and expectations of AMRs.

Finally, the relationship between standards and terminology is not purely top-down. Interoperability initiatives often succeed by producing implementable interfaces and shared semantics. They can also shape what stakeholders mean by a term because the working groups shaping the concept in concrete message sets, behaviors and integration practices. The VDA5050 is a successful example of how communication between vehicles and fleet management can be standardized. The name is derived from the German abbreviation of the German Association of the Automotive Industry (VDA, Verband der Automobilindustrie e.V.) and the document number. The protocol is now used by companies worldwide (Tomatis, 2021). However, the working group’s efforts have also influenced the terms and vehicles to which the protocol applies. e.g., the first public version 1.1 still refers to AGVs and driverless transport systems, while the latest version 3.0 only refers to MRs (VDA 5050 Community, 2026). Similarly, the MassRobotics AMR Interoperability Standard enables AMR and autonomous vehicles to share various information with each other from multiple vendors and have them work together in the same environment. Other vehicle types are not mentioned (MassRobotics, 2021). In addition, there are other initiatives worldwide that are attempting to standardize creating open protocols and documentation (Franke et al., 2026b). In doing so, they use terms without explaining them further or providing a reference such as ISO (Franke et al., 2023). From a terminology perspective, these initiatives matter because they provide shared operational reference points (e.g., what fleet manager, task or vehicle state entails), which can stabilize or shift how practitioners interpret labels such as AGV and AMR. In other words, even when vocabulary standards define terms or safety standards acknowledge multiple coexisting names, interoperability ecosystems can still influence terminology from within by establishing the meanings that stakeholders enact during implementation and deployment.

In addition to formal standards, the academic literature routinely introduces its own working definitions of AMR and MRs (Franke et al., 2026a). They are often tailored to the paper’s research focus (e.g., navigation, planning/control, safety, or application domain). Recent review work in warehousing explicitly frames an evolution from AGVs to AMRs and differentiates the terms along capability axes such as infrastructure dependence and autonomy: *“[…] AGVs can only move on fixed paths”* while *“[…] AMRs can move autonomously, collision-free and navigate without additional infrastructure within a given area to any accessible target point using onboard laser scanners”* (Lackner et al., 2024). Similarly, operations-research oriented reviews define AMRs through decision-making and control characteristics, contrasting AMRs with AGV systems *“[…] in which a central unit takes control”* whereas AMRs can *“[…] communicate and negotiate independently with other resources like machines and systems and thus decentralize the decision-making process”* (Fragapane et al., 2021). Even outside warehousing, broader surveys adopt pragmatic definitions tied to navigation and supervision, e.g., describing AGVs as relying on *“[…] tracks or predefined routes”* while AMRs are *“[…] an evolution of AGVs”* that can *“[…] move independently in their environment”* (Zhang et al., 2023). These examples illustrate that academic definitions often converge on a small set of recurring dimensions, with nuances in technical details, reinforcing the need to empirically examine how experts actually interpret and apply the labels in practice.

Table 1 summarizes the standards, regulations, initiatives and academic sources referenced in this Subsection. The Table provides an overview of where terminology is defined versus merely mentioned or used in practice and which terms (MR, AGV, AMR, DTV) are covered.

**TABLE 1:** Overview of standards, initiatives and academic sources relevant to terminology in industrial mobile robotics.

Reference and name	Landscape	Term coverage	Terminology reference
ISO8373 International Organization for Standardization, (2021)	Standard (vocabulary)	MR: D; AGV: M; /D AMR: —; DTV: —	Defines MR and related core terms; broad terminology
ISO19649:2017 International Organization for Standardization, (2017)	Standard (vocabulary)	MR: M/D; AGV: —; AMR: —; DTV: —	Applies MR vocabulary consistent with ISO8373
ISO3691-4 International Organization for Standardization, (2023)	Standard (safety)	MR: —; AGV: M; AMR: M; DTV: —	Frames safety scope and acknowledges multiple coexisting labels (e.g., AGV/AMR) for driverless systems
ISO5053-1 International Organization for Standardization, (2020)	Standard (vocabulary)	MR: —; AGV: —; AMR: —; DTV: —	Defines industrial truck terminology referenced by safety standards; only mentions driverless trucks
VDI2510 Verein Deutscher Ingenieure, (2005)	Guideline (domain practice)	MR: —; AGV: D; AMR: —; DTV: D (in German)	Defines AGVs in the context of AGV systems; influential in German-speaking practice (published bilingual)
DTV usage examples Hansen and Gillert (2008); Arvato (2020); BMW Group (2024); Ehrhardt Partner Group, (2026)	Practice (industry)	MR: —; AGV: —; AMR: —; DTV: M/D	Illustrates DTV as a practice term used in English-language communication, often in translation contexts
ISO/DIS21423 International Organization for Standardization (2026c)	Standard (draft)	MR: —; AGV: —; AMR: M; DTV: —	Frames AMR systems and interoperability interfaces; term usage likely shapes shared interpretations even without strict definitions
ANSI/RIA R15.08-1-2020 Institute (2020)	Standard (safety, US)	MR: —; AGV: D; AMR: D; DTV: —	Provides operational definitions differentiating AGV and AMR primarily via navigation/path generation
Regulation (EU) 2023/1230 European Parliament and Council of the European Union, (2023)	Regulation (safety, EU)	MR: —; AGV: —; AMR: (M) (as autonomous mobile machinery); DTV: —	Introduces requirements for autonomous mobile machines; may indirectly shape expectations and perceived requirements for AMR-class systems
VDA5050 VDA 5050 Community (2026)	Industry initiative from Germany (interoperability)	MR: M; AGV: M; AMR: —; DTV: —	Interface recommendation; term usage evolved across versions (e.g., AGV/DTS to MR), affecting community terminology in practice
MassRobotics AMR interoperability Standard MassRobotics (2021)	Industry initiative from US (interoperability)	MR: —; AGV: —; AMR: M; DTV: —	Uses AMR as the primary category for multi-vendor interoperability
Academic working definitions (examples) Lackner et al. (2024); Fragapane et al. (2021); Zhang et al. (2023)	Academic literature	MR: M/D; AGV: M/D; AMR: M/D; DTV: —	Introduces context-specific working definitions (navigation, control, autonomy, application)

Term coverage indicates whether a term is defined (D), mentioned/used (M), or not covered (−).

Table 1 highlights a persistent gap between normative and lived terminology in industrial MRs. The epistemic difference is not clear from the classic available definitions, so the communities have come up with their own creations or additions. As a result, the definitional rule of clarity is not consistently met in practice. Stakeholders introduce additional labels, translate terms, or adopt context-dependent criteria when classical definitions do not lead to a perceivable conceptual difference. This motivates an empirical perspective. Instead of proposing yet another definition, our survey captures how experts actually perceive and use these terms. Covering spontaneous associations, perceived separability, agreement with terminology statements and the attribution of technical characteristics thereby revealing where current standardization aligns with practice and where communication relevant gaps remain.

## 3 Methods

To investigate how experts interpret and distinguish terminology in industrial MRs, we conducted an online survey. The subject of the investigation is the set of terms AGV, AMR, DTV, and MR. Industrial trucks are excluded due to the large number of manually operated vehicles. Also the term mobile manipulator is excluded since the term is not part of the relevant standards discussed in Section 2. Other widely used terms, such as unmanned ground vehicle (UGV) or its counterpart, unmanned aerial vehicle (UAV), are not included in the investigation of interpretation of the term characteristics. The term UAV appears only one time in Question 9, where participants are asked to classify related emerging technologies in the context of the main investigation. UGVs are primarily used in military or public sectors (Council et al., 2003; Ni et al., 2021). Since UAVs are flying robots, their use in industry is already possible but is severely restricted by regulations (Stöcker et al., 2017). These robot categories are not listed on the websites of major industrial robot manufacturers, are not included in standardization documents in an industrial context, and are not contained in databases such as the ISO Online Browsing Platform (International Organization for Standardization, 2026a). This also applies to broader terms such as autonomous vehicles, which appear in standardization documents in the automotive sector but not in the industrial sector, for example, ISO37168 and ISO22133 (International Organization for Standardization, 2022; International Organization for Standardization, 2026b).

A survey was chosen for three reasons. First, the raised questions address both associations (e.g., which concepts are spontaneously linked to a term) and structured judgments (e.g., perceived distinguishability, agreement with general statements and attribute assignments), which can be systematically elicited with standardized items. Second, the survey format enables consistent questioning across participants with diverse roles (e.g., research, development and management) and supports subgroup comparisons under identical item wording. Third, the study aims to capture perceptions as they occur in professional communication. Therefore, several items were designed to elicit mental models rather than formal definitions by prompting spontaneous responses and practical classification decisions.

Participation in the survey was voluntary and anonymous. Although the broader robotics and automation community is large, combinations of demographic variables may still act as quasi-identifiers in a small expert sample. Therefore, no directly identifying personal data were collected and results are reported in aggregated form, but complete anonymization cannot be guaranteed in a strict technical sense. Before starting the survey, participants were informed about the purpose of the study. No identifying personal data were collected. Participants could skip individual questions and terminate the survey at any time without consequences.

### 3.1 Survey and participants

The survey included demographic questions (Question 1 to Question 4) followed by content questions on term perception (Question 5 onwards). All survey questions and response formats are provided in the Supplementary Material. The survey combined complementary question types to capture different facets of terminology perception. It was designed as an exploratory instrument and intentionally combines heterogeneous question formats, including free associations, distinguishability ratings, classification tasks, and attitude statements. These items address different aspects of terminology perception and were therefore not intended to form a single latent construct.

To ensure that the survey captures informed and practice-relevant perspectives, participants were recruited using a targeted expert sampling approach. The authors disseminated the survey through their professional networks, LinkedIn and direct interactions during a trade fair in the field of warehousing and robotics. This combination of channels was chosen to deliberately reach professionals with direct experience in the planning, development, deployment, or operation of robotic systems, rather than a general audience. As a result, the sample reflects a focused expert group relevant to the research gap addressed in this study. The group is therefore not statistically representative, but is intended to specifically examine the opinions of experts in the field of warehousing and thus also likely to contain a sampling bias. Consequently, the sample should not be considered representative of the broader industrial mobile robotics community. The findings therefore primarily reflect the perceptions of a specific expert population and should be interpreted as exploratory rather than generalizable to all stakeholder groups. A total of 53 experts completed the survey. The survey consisted of 13 questions and was offered in German and English. Data were collected between 19 August 2025 and 14 October 2025. In total, 309 responses were recorded, 53 were complete and were included for analysis, while 256 responses were incomplete and excluded. No answers from the incomplete responses were saved, so they are not suitable for further analysis. In the following, therefore, only the complete responses will be analyzed and discussed.

The first four questions asked about the demographic characteristics of the participants, including their professional role, the functional area, their age and the country in which they work. The roles and functions from Question 1 and Question 2 are derived from the relevant literature (Schramm, 2020).

Most participants identified themselves as skilled workers or specialists without management responsibility (n=29) . In addition, 19 participants reported working in departmental, team, or group management roles, while 3 participants belonged to executive or division management. One participant identified as a trainee or working student and one participant reported a role in chief executive management. Two respondents selected “other” based on their free-text descriptions, both were assigned to the category of skilled workers because the description in the free text field under other indicated the category skilled worker. Overall, the sample is thus dominated by operational and mid-level decision-making roles.

The majority of participants worked in research and development (n=36) . Further respondents were active in service creation or production (n=7) , followed by distribution or sales (n=4) , marketing or communication (n=3) , purchasing or procurement (n=2) and customer service or support (n=1) . This distribution reflects a major research background within the sample.

Participants’ ages ranged from 25 to 64 years, with one outlier value of 99, which is likely due to intentional non-disclosure. Including all responses, the mean age was 38.11 years (median age 38 years); excluding the outlier, the mean age was 36.94 years (median age 37 years). The age distribution indicates a predominantly mid-career expert sample with substantial professional experience.

Most participants were based in Germany (n=50) , with additional respondents from Austria (n=2) and the United States (n=1) . The sample therefore primarily reflects perspectives from the German-speaking industry and academia due to the sample.

### 3.2 Statistical analysis methods

In addition to classical descriptive measures (mean, median, mode), more advanced modeling and statistical inference procedures are applied to selected survey questions. The rationale for their application, as well as the exact methods and procedures, are described in the following paragraphs. Most of the outcome variables are measured on an ordinal scale (Likert-type). Although Likert-type items produce ordinal data and the subjective distance between adjacent response categories cannot be assumed to be strictly equal, they represent an established and widely used instrument for capturing attitudes, perceptions, and self-assessments in survey research (Sullivan and Artino Jr, 2013; Jebb et al., 2021). In the present study, this format was therefore used to elicit structured and comparable expert judgments on terminology perception, while the ordinal nature of the data was accounted for by reporting medians and by using rank-based nonparametric procedures for statistical inference. For descriptive and exploratory purposes, mean values are reported in addition to medians and modes. This practice is common in survey research and implicitly assumes an approximately interval-scaled interpretation of the response categories. These robust and nonparametric methods do not rely on assumptions of normality of the response variable.

All analyses are conducted using the software R (Version 4.5.2) (R Core Team, 2025). Statistical tests and confidence intervals (CIs) are performed at the conventional significance level of α=0.05 (corresponding to 95% CI coverage). Throughout this paper, the term “statistically significant” refers to this significance level. As no hypotheses or analysis plans were preregistered, the analyses are intended to be exploratory in nature and should be interpreted as hypothesis-generating. Therefore, no formal adjustment for multiple testing was applied. Consequently, statistically significant findings should be interpreted as indications of potentially relevant patterns rather than confirmatory evidence.

Analysis Method for Question 6. A particular substantive focus lies on the results of Question 6. In this question, participants were asked to rate their perceived ability to conceptually distinguish between all different pairs of the four investigated terms on a five-point Likert scale.

Given the pivotal role of these self-assessments for the substantive interpretation of the study, the underlying data structure is formally modeled and subjected to inferential statistical analysis to determine whether the observed patterns reflect systematic effects rather than random variation. Each participant provides six ordinal repeated measurements. These six responses correspond to all possible pairwise comparisons between the four investigated terms. For this fully crossed repeated-measures design with ordinal, and therefore non-normal outcomes, a rank-based hierarchical data model with one within-subject factor is performed by applying the function nparLD from the R package of the same name (Noguchi et al., 2012). This approach provides a robust alternative to a classical repeated-measures ANOVA (Analysis of Variance), which may yield invalid results when assumptions such as normality are violated.

To explain this explicitly, let $Y_{ij}$ denote the ordinal response of participant i for robot pairing j , with j∈{1,…,6} and i∈{1,…,53} . The nonparametric target quantities in this model are the so-called relative effects (REs), which quantify the probability that an observation from a given condition is larger than a randomly selected observation from the pooled distribution of all conditions. The RE for pairing j , is defined as

$$p_{j}=P\left(Y_{ij}>Y_{\cdot }\right)+0.5\cdot P\left(Y_{ij}=Y_{\cdot }\right),$$ (1)

where for each pairing j , the responses are assumed i.i.d. across participants with marginal distribution $F_{j}s$ , and $Y_{\cdot }$ denotes the outcome variable of an independent, randomly selected pairing, i.e., $Y_{\cdot }$ is a random draw from the equally weighted mixture of the $F_{j}$ .

Based on these effects, an ANOVA-type statistic is used to assess the global hypothesis

$$H_{0}:F_{1}=\ldots =F_{6}.$$ (2)

The hypothesis states that there are no differences in the distributions of the response variable across the pairings (see e.g., Akritas et al., 1997). Rejection of this hypothesis indicates that the response distributions differ, suggesting that the patterns observed in the heatmap reflect genuine distributional differences. Trends in the individual pairing distributions are assessed by examining the estimated REs and corresponding confidence intervals (CIs, for their construction within nparLD, see Brunner et al. (2002)). Statistical significance of individual tendencies is concluded when the corresponding CI does not include 0.5. By construction of the RE in Equation 1, this value represents a neutral effect.

Additionally, we visualized the pairwise RE values on a heat map. To enhance comparability with the correlation analysis building on Question 8 (see next paragraph), we rescaled the RE values to the domain [−1,1] via $2\;p_{j}-1$ . This is a common transformation for similar quantities, which additionally changes the interpretation such that negative values indicate lower distinguishability of the pairing, and positive values indicate higher distinguishability (see, e.g., Buyse et al., 2025).

Analysis Method for Question 8. Building upon Question 6, Question 8 is analyzed by comparing the participants’ self-assessments with the actual assignment of robot characteristics. Thirteen attribute statements are provided, which participants assign to the four robot types. The results are presented in a visualization showing the average selection proportion per statement and robot in a 13×4 matrix. Rare selections (down to 0%) are displayed in white, while frequent selections (up to 100%) are displayed in blue. Marginal proportions are also reported (bottom row and rightmost column), indicating how often a statement was selected on average across all robots and how many statements were selected on average for each robot.

To examine whether the distinguishability based on assigned attributes corresponds to the self-assessments from Question 6, rank correlations, specifically Kendall’s τ , are computed using the average assignment proportions across robot types. Graphically, these correspond to the correlations between the columns of the previously described result matrix. A low correlation (minimum −1 ) indicates high distinguishability, whereas a high correlation (maximum +1 ) indicates low distinguishability. To facilitate comparison with Question 6, an equivalent heatmap is constructed. To ensure conceptual consistency of the color scale, negative correlations are displayed in green and positive correlations in red.

Analysis Method for Question 7 and 10. A central aspect of this study is to investigate potential differences between participant groups. Group differentiation is based, first, on Question 1, distinguishing Management (response options 1–3) and non-Management (response options 4–6), and second, on Question 2, distinguishing Research and Development (R&D; response option 8) and non-R&D (response options 1–7). All groups contain sufficient sample sizes (Management n=23 , non-Management n=30 (29 skilled worker plus 1 trainee); R&D n=36 , non-R&D n=17 ). The outcome variables from Questions 7 and 10 are also of Likert type. Question 7 comprises seven statements, and Question 10 comprises eight statements. To assess whether the distributions differ between the respective groups, a permutized version of the Brunner–Munzel (BM) test is applied (Brunner and Munzel, 2000; Pauly et al., 2016). Analogous to the model used for Question 6, the BM test is a nonparametric, rank-based procedure. The effect measure of interest for each pairwise comparison is defined as

$$\tilde{p}=P\left(Y_{1}>Y_{2}\right)+0.5\cdot P\left(Y_{1}=Y_{2}\right),$$ (3)

which quantifies the probability that a randomly selected observation from the first group $\left(Y_{1}\right)$ exceeds a randomly selected observation from the second group $\left(Y_{2}\right)$ . In contrast to the model used for Question 6, $Y_{1}$ and $Y_{2}$ are independent here, as they reflect answers from different participants. For a two-sided test, the null hypothesis is given by $H_{0}:\tilde{p}=0.5$ , assessing whether the outcomes of the two groups are stochastically equivalent. In contrast to the Mann–Whitney–U test, the BM test remains valid under unequal variances and differing distributional shapes between groups. In addition, the permuted version constructs the null distribution of the test statistic empirically via repeated permutations of the group labels. This avoids reliance on the asymptotic distributional approximation of the BM test statistic and can improve the accuracy of the resulting p-values, particularly in small samples.

Since the items do not constitute a unified measurement scale but rather capture largely independent research dimensions, they are analyzed individually. No formal multiplicity adjustment is applied due to the exploratory character of the analysis. Consequently, statistical significance is interpreted with caution. The result tables in the following section report the p -values of the BM tests. Values below 0.05 are marked as statistically significant. The corresponding CIs are described in the text. The permuted BM tests were conducted using 9,999 permutations with the R package TOSTER (Lakens, 2017).

## 4 Results and discussion

This section presents the results of the conducted survey and discusses them in the context of the perception of DTVs, AGVs, AMRs and MRs. First, the empirical findings are reported in a descriptive manner. Subsequently, these results are discussed and interpreted with respect to the research objectives and existing literature.

The first question (Question 5 for the participants) regarding the perception of AGVs, AMRs, DTVs and MRs included a free-association task. Participants were asked to provide up to three spontaneous terms for each of the four target terminologies. The aim of this question was not to assess compliance with formal definitions, but to capture salient associations that shape expectations and communication in professional contexts. All responses were coded into six categories reflecting recurring themes.

1. Navigation & Motion,
2. Locus of Control,
3. Autonomy & Capabilities,
4. Judgmental Properties,
5. Application/Context and
6. Terminology/Culture/Standards.

Table 2 summarizes the resulting category frequencies per term.

**TABLE 2:** Question 5: Category frequencies from the free-association task (counts per term).

Category	DTV	AGV	AMR	MR
1. Navigation & motion	31	36	24	5
2. Locus of control	7	11	17	6
3. Autonomy & capabilities	33	28	32	34
4. Judgmental properties	19	12	37	28
5. Application/Context	10	5	5	4
6. Terminology/Culture/Standards	30	31	14	36

Overall, the patterns indicate distinct and term-specific mental models. For DTV and AGV, associations were strongly dominated by Navigation & Motion, Autonomy & Capabilities as well as Terminology/Culture/Standards, suggesting that these terms are primarily linked to route-constrained motion concepts and to established naming conventions or standard-related interpretations. DTV and AGV are often associated with the application in warehousing. In contrast, AMR is characterized by a pronounced emphasis on the categories Judgmental Properties and Autonomy & Capabilities, indicating that experts commonly connect AMRs with modernity, flexibility and higher autonomy. The Locus of Control of AMRs is connected to decentralized concepts. MR shows the strongest concentrations in Autonomy & Capabilities and Terminology/Culture/Standards, accompanied by comparatively low counts for Navigation & Motion and Application/Context. This distribution is consistent with MR being used as a broad concept that evokes general capability expectations and definitional ambiguity rather than a specific operational or application-centered image.

Taken together, the free-association results suggest that the four terms are not merely technical labels but carry systematically different connotations regarding motion concepts, control assumptions, perceived capability profiles and the role of standards and language. This supports the view that terminology can actively shape expert expectations and therefore affects communication across stakeholders.

Question 6 assessed whether experts perceive the investigated terms as conceptually separable. Participants rated the distinguishability of six term pairs on a five-point Likert scale ranging from 1 (“not distinguishable/separable at all”) to 5 (“clearly distinguishable/separable”). The item was designed to capture perceived conceptual boundaries between frequently used labels (DTV, AGV, AMR and MR), independent of whether respondents adhere to any specific standard definition.

**FIGURE 1:**
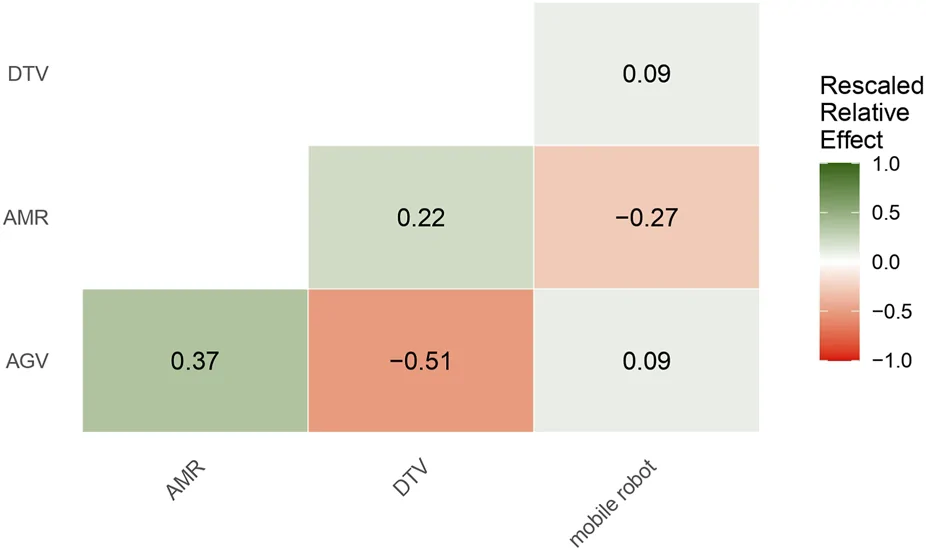
Question 6: Heatmap of the rescaled relative effects (REs) for each pair of terms: DTV (Driverless Transport Vehicle), AGV (Automated Guided Vehicle), AMR (Autonomous Mobile Robot), and MR (Mobile Robot). Values range from −1 (red), indicating lower perceived distinguishability, to +1 (green), indicating higher perceived distinguishability.

Figure 1 provides a visual overview of the self-assessed distinguishability ratings. The heatmap displays the pairwise rescaled REs from −1 = not distinguishable to +1 = clearly distinguishable and therefore highlights descriptive tendencies at a glance. The strongest positive contrast is visible for the pair AGV–AMR, whereas AGV–DTV shows the strongest negative value, indicating that respondents perceived these two terms as least distinguishable. The pairs AGV–MR and DTV–MR lie close to 0, suggesting only moderate separability, while AMR–MR is shifted toward the non-distinguishable side. AMR–DTV is rated on the distinguishable side, but less strongly than AGV–AMR. The same tendencies are reported numerically in Table 3, which additionally includes estimated relative effects (REs) together with 95% confidence intervals.

**TABLE 3:** Results for question 6: Perceived distinguishability of DTV (Driverless Transport Vehicle), AGV (Automated Guided Vehicle), AMR (Autonomous Mobile Robot) and MR (Mobile Robot).

Term pair	Mean	Median	Mode	Estimated RE	95% CI for RE
AGV and AMR	3.81	4	4	0.69	[0.63, 0.73] (*)
AGV and DTV	1.62	1	1	0.25	[0.20, 0.31] (*)
AMR and DTV	3.43	4	5	0.61	[0.55, 0.66] (*)
AMR and MR	2.21	2	2	0.37	[0.32, 0.41] (*)
AGV and MR	3.11	3	3	0.55	[0.50, 0.59]
DTV and MR	3.09	3	3	0.54	[0.49, 0.59]

Mean, median, mode are reported, along with RE estimates and 95% CIs; (*) indicates REs significantly different from 0.5.

Table 3 summarizes the inferential and descriptive results. The estimated REs indicate clear differences in perceived distinguishability between the term pairs. AGV–AMR (RE = 0.69) and AMR–DTV (RE = 0.61) are significantly above 0.5, suggesting that participants generally perceived these pairs as distinguishable. In contrast, AGV–DTV (RE = 0.25) and AMR–MR (RE = 0.37) are significantly below 0.5, indicating substantial perceived overlap between these terms. The REs for AGV–MR and DTV–MR are close to 0.5. This suggests that the responses do not show a clear directional tendency toward either strong separability or strong overlap for these two pairings.

The descriptive statistics are consistent with these findings. The highest perceived distinguishability was reported for AGV and AMR (mean = 3.81; median = 4; mode = 4), followed by AMR and DTV (mean = 3.43; median = 4; mode = 5). In contrast, AGV and DTV showed the lowest perceived distinguishability (mean = 1.62; median = 1; mode = 1), indicating that respondents largely treated these terms as interchangeable or as describing strongly overlapping concepts. AMR and MR also showed limited separability (mean = 2.21; median = 2; mode = 2). The pairings AGV–MR (mean = 3.11) and DTV–MR (mean = 3.09) were positioned near the midpoint of the scale, which is consistent with the absence of a clear directional tendency in the corresponding RE estimates.

These findings support two key interpretations. First, the near-non-distinguishability of AGV and DTV suggests that DTV is widely perceived as a synonym or variant of AGV rather than as a distinct category, which suits the translation. Second, the comparatively high distinguishability of AGV and AMR indicates that experts hold a salient conceptual contrast between these labels, even though the boundaries are not universally agreed upon.

Question 7 assessed experts’ agreement with a set of general statements about the relationship and usefulness of the terms DTV, AGV, AMR and MR. All statements were rated on a five-point Likert scale (1 = disagree completely, 5 = agree completely). In addition to reporting the overall tendencies, we differentiated responses by professional background to explore whether perceptions vary across stakeholder groups. Group assignment was derived from participants’ functional area (Question 2), separating respondents working in R&D from non-R&D roles and second management versus non-management positions derived from Question 1.

Table 4 summarizes the results for the overall sample and the group comparisons including BM p -values for both pairwise comparisons. Across statements, mean values are generally close to the neutral midpoint, indicating that participants hold nuanced rather than polarized views. At the same time, three broader patterns emerge. First, respondents tend to endorse a hierarchical framing in which DTV, AGV and AMR can be understood as subcategories of MRs (overall mean = 3.79), with R&D respondents showing significantly stronger agreement than non-R&D (4.11 and 3.12, BM- p<0.005 ). The corresponding CI for this statement is entirely above value of 0.5 (95% CI: [0.593, 0.879]), indicating a robust tendency toward higher agreement in the R&D group. This indicates that respondents with a higher technical/development background are more likely to apply an explicit taxonomy in which MR can be used as a broad category. Second, views on the professional adequacy of the term MR are mixed: the sample shows only moderate agreement that the term is too vague (mean overall 3.13) and slightly lower agreement that it is sufficient (mean overall 2.96). Third, respondents generally reject the notion that the distinctions are irrelevant to their work (mean overall 2.09) and tend to disagree that the term AGVS (Automated Guided Vehicle System) should designate individual vehicles (mean overall 2.42), indicating that terminology is perceived as practically consequential and that system-level and vehicle-level labels are not considered interchangeable.

**TABLE 4:** Results of question 7: Agreement with general statements on DTV (Driverless Transport Vehicle), AGV (Automated Guided Vehicle), AMR (Autonomous Mobile Robot) and MR (Mobile Robot) with differentiation in the whole group (overall), R&D and Management (Mgmt); as well as the p -values of the permuted-BM test for both pairwise group comparisons.

Statement	Mean	Median	Mode	Mean	Mean	BM	Mean	Mean	BM
	Overall	Overall	Overall	R&D	Non-R&D	p -val	Mgmt	non Mgmt	p -val
The terms (DTV, AGV and AMR) are subcategories of mobile robots	3.79	4	5	4.11	3.12	0.005(*)	3.96	3.67	0.305
The term mobile robot is too vague for professional use	3.13	3	4	3.00	3.41	0.337	2.83	3.37	0.163
The term mobile robot is sufficient for professional use	2.96	2	2	3.11	2.65	0.218	3.09	2.87	0.545
For professional use, further abstraction can be applied and terms such as robot or carriage can be used	3.06	3	4	3.19	2.76	0.231	3.09	3.03	0.699
The terms depend heavily on the application	3.53	4	4	3.64	3.29	0.422	3.22	3.77	0.106
The distinction is not relevant to my work	2.09	2	1	2.14	2.00	0.340	2.43	1.83	0.145
The term AGVS (automated guided vehicle system) can be used to designate individual vehicles	2.42	2	1	2.42	2.41	0.881	2.52	2.33	0.611

(*) indicating significant results.

Comparing groups, differences between management and non-management respondents are small, suggesting broadly shared perceptions across roles in this sample. Consistent with this, the CIs for the management comparison overlap 0.5 for all statements (95% CIs: [0.422, 0.736] to [0.222, 0.527]), indicating no clear directional differences between management and non-management. In contrast, the R&D vs. non-R&D split reveals one meaningful divergence: R&D respondents more strongly support the conceptual inclusion of DTV, AGV, AMR under MRs and this is the only statistically significant group difference in Question 7 (BM- p=0.005 ). All remaining R&D CIs overlap 0.5 (95% CIs: [0.236, 0.601] to [0.328, 0.698]), suggesting no consistent subgroup differences beyond Statement 1. Overall, the results indicate partial consensus on the need for structured terminology, while simultaneously highlighting that the term MR remains contested in terms of its professional precision and practical communicability.

Question 8 examined which technical characteristics experts assign to the investigated terms. The item was implemented as a multiple-choice matrix in which participants could select one, several, or none of the terms (DTV, AGV, AMR, MR) for each technical statement. To summarize the results, we report selection proportions across all participants, indicating how frequently a given attribute was assigned to each term.

**FIGURE 2:**
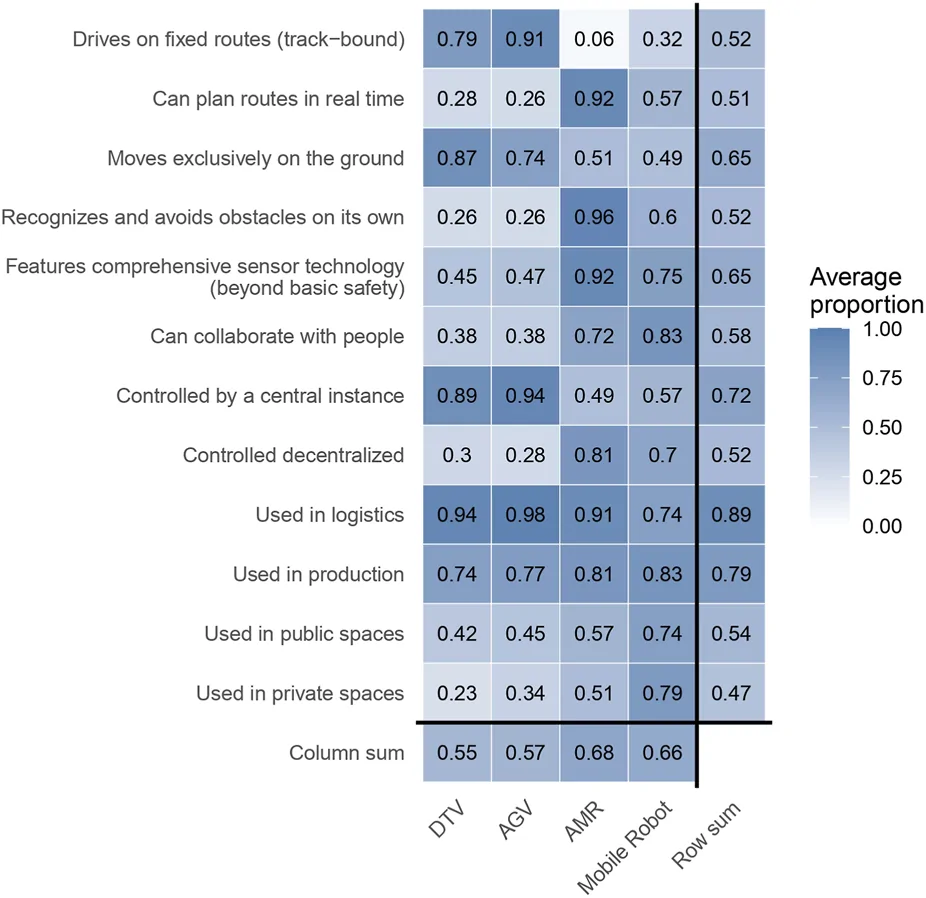
Results of question 8: Selection proportions per statement and vehicle (DTV (Driverless Transport Vehicle), AGV (Automated Guided Vehicle), AMR (Autonomous Mobile Robot) and MR (Mobile Robot)), ranging from 0 (never selected, shown in white) to 1 (always selected, shown in blue).

**FIGURE 3:**
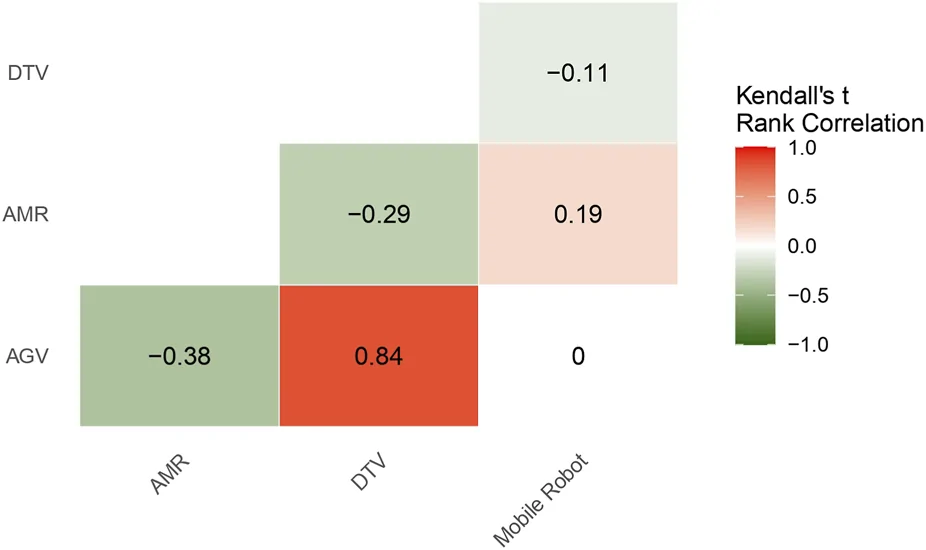
Question 8: Heatmap of Kendall’s rank correlations (τ) between the attribute-selection profiles for each pair of terms: DTV (Driverless Transport Vehicle), AGV (Automated Guided Vehicle), AMR (Autonomous Mobile Robot), and MR (Mobile Robot). Values range from −1 (green), indicating lower similarity and thus higher distinguishability, to +1 (red), indicating higher similarity and thus lower distinguishability. The color scale is aligned conceptually with Figure 1 from Question 6.

Figure 2 provides the attribute-level view of the results. It shows that DTV and AGV are assigned highly similar technical profiles, while AMR and MR are associated with partly distinct capability patterns. To complement this statement-level perspective, Figure 3 visualizes the pairwise rank correlations (Kendall’s τ ) between the complete attribute profiles of the four terms. The heatmap therefore serves as a compact similarity map of expert expectations across all technical characteristics, confirming the expectations from Question 6, which can be compared in Figure 1.

The correlation heatmap in Figure 3 reveals one dominant pattern: DTV and AGV show a very high positive correlation, indicating that experts assign very similar technical characteristics to both terms. This strongly supports the interpretation that both labels evoke nearly the same technical mental model in practice. By contrast, the correlations between AGV/DTV and AMR are clearly lower and even negative, reflecting a systematic contrast in the attributed properties. This confirms that participants do not merely use AMR as a modern synonym, but associate it with a distinctly different capability profile. The correlations involving MR are weaker and more mixed, which is consistent with MR being used as a broader and less sharply delimited term.

At the level of individual attributes (Figure 2), both DTV and AGV receive high selection proportions for driving on fixed routes, moving exclusively on the ground, being centrally controlled, and being used in logistics. The largest difference between both terms appears for the statement “drives on fixed routes (track-bound)” where AGV is selected more often than DTV. This suggests that DTV is perceived as slightly less strictly tied to route-bound navigation, despite the overall similarity of both terms.

AMR is characterized by a markedly different profile. Experts frequently associate AMR with real-time route planning and explicitly do not associate it with fixed route sequences. In addition, AMR is linked to autonomous obstacle detection and avoidance, comprehensive sensor technology, and decentralized control. These assignments align with the free-association results from Question 5, where AMR was also connected to autonomy, flexibility, and decentralized operation. The area of application refers to logistics and production.

The term MR shows a broader and less specific profile than DTV, AGV, and AMR. Selection proportions are generally more moderate, and no single technical attribute dominates as strongly as for AGV or AMR. The highest values are observed for collaboration with humans and use in production, while MR also receives comparatively high selections for public and private spaces. Together with the weaker correlation pattern, this indicates that MR functions as a broad category label that can accommodate heterogeneous systems, but does not communicate a sharply defined technical configuration on its own.

Overall, Question 8 reinforces the central finding of this study that terminology in industrial mobile robotics is associated with distinct expectation profiles. In particular, AGV and DTV are perceived as technically close but not identical, whereas AMR is associated with a more autonomous and decentralized capability profile. MR, in contrast, is used more broadly and remains less specific in the technical expectations it evokes.

Question 9 examined how experts conceptually place adjacent robotic technologies relative to the four target terms. Participants were asked to classify cobots (collaborative robots), drones/UAVs (unmanned aerial vehicles) and humanoid robots by selecting one or multiple categories (part of MRs, AGVs, DTVs, AMRs, own class, or I do not know). This multiple-choice format was chosen deliberately because these technologies may reasonably be understood either as subclasses of existing categories or as distinct classes with only partial overlap, depending on respondents’ assumptions.

Table 5 shows that respondents most often treat all three technologies as their own class, particularly for drones/UAVs (38 mentions) and humanoid robots (37 mentions). Cobots are identified as their own class by 25 participants. At the same time, both humanoid robots and cobots are frequently classified as part of MRs (24 mentions each), indicating that a substantial subset of experts interprets MR as a broad term that can include diverse embodiments beyond wheeled transport platforms. The term humanoid was examined solely for classification purposes, as no established industrial application had been identified at the time of the study Eßer et al. (2026). Cobots exhibit the most divided classification: while many respondents assign them to an own class and to MRs, they are also relatively often linked to AMRs (14 times), suggesting that some experts implicitly associate collaborative robotic functionality with mobile platforms or with the AMR concept in particular. In contrast, only small numbers classify any of the three technologies as part of AGVs or DTVs, reinforcing the view that these terms are perceived as narrower, logistics-centered categories rather than general descriptors for robotic mobility.

**TABLE 5:** Results of question 9: Total counts of classification of related technologies (Cobots, Drones/UAVs and Humanoids) in the context of investigated terms.

Technology	Part of mobile robots	Part of AGVs	Part of DTVs	Part of AMRs	Own class	I do not Know
Cobots (collaborative robots)	24	4	2	14	25	5
Drones/UAVs (unmanned aerial vehicles)	16	4	2	8	38	2
Humanoid robots	24	1	1	6	37	1

Overall, these results support two interpretations. First, the high frequency of own class selections for drones/UAVs and humanoid robots indicates that experts maintain clear conceptual boundaries for technologies that differ strongly in locomotion mode and appearance. Second, the parallel tendency to classify cobots and humanoid robots as part of MRs suggests that MR is used as a high-level abstraction, which can accommodate heterogeneous systems, potentially explaining why this broad term is simultaneously seen as both useful and ambiguous in professional terminology.

**FIGURE 4:**
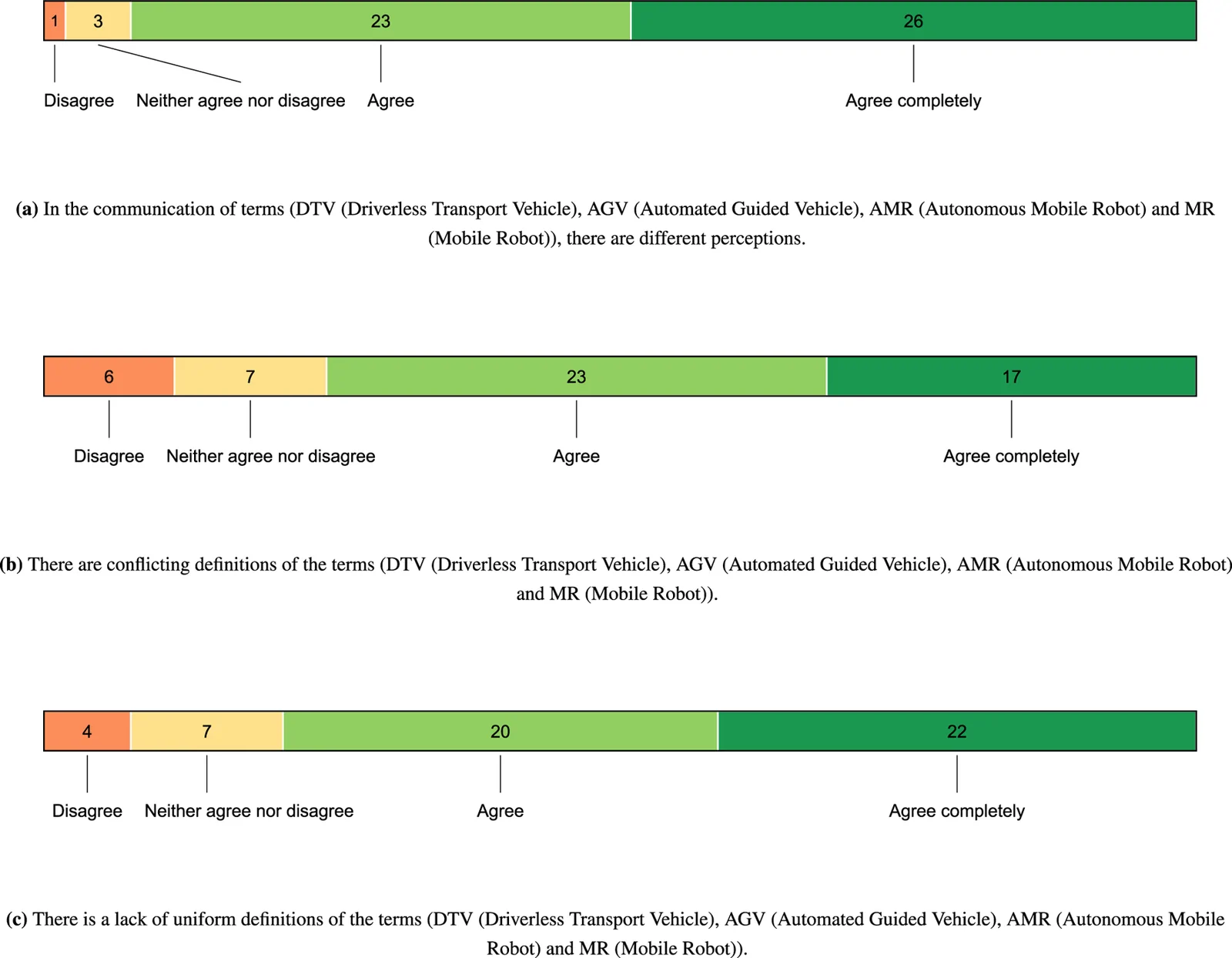
Results for question 10 (Likert distributions). **(a)** Different perceptions in term communication. **(b)** Conflicting definitions of the terms. **(c)** Lack of uniform definitions. None of the participants selected the option Disagree completely in all three questions.

Question 10 examined how experts perceive the current state of definitions and standardization for the terms DTV, AGV, AMR and MR. Participants rated eight statements on a five-point Likert scale (1 = disagree completely, 5 = agree completely). Beyond the overall tendencies, we again differentiated responses by professional background to test whether perceptions vary systematically across stakeholder groups. Table 6 summarizes the results, including BM p -values for both pairwise group comparisons. In addition, we visualize the response distributions for the first three statements in Figure 4 to highlight the strongest signals motivating this work.

**TABLE 6:** Results of question 10: Perception of definitions and standards for DTV (Driverless Transport Vehicle), AGV (Automated Guided Vehicle), AMR (Autonomous Mobile Robot) and MR (Mobile Robot) with differentiation in the whole group (overall), R&D and Management (Mgmt), as well as the BM- p -value for both pairwise group comparisons.

Statement	Mean overall	Median overall	Mode overall	Mean R&D	Mean Non-R&D	BM	Mean	Mean	BM
						p -val	Mgmt	non Mgmt	p -val
In the communication of terms (DTV, AGV, AMR, mobile robot), there are different perceptions	4.40	4	5	4.44	4.29	0.247	4.26	4.50	0.241
There are conflicting definitions of the terms (DTV, AGV, AMR, mobile robot)	3.96	4	4	3.83	4.24	0.132	3.70	4.17	0.094
There is a lack of uniform definitions of the terms	4.13	4	5	4.08	4.24	0.527	3.87	4.33	0.063
Existing definitions are outdated	3.40	3	3	3.33	3.53	0.324	3.39	3.40	0.904
There is no suitable definition for the term DTV yet.	2.58	3	3	2.78	2.18	0.047(*)	2.30	2.80	0.096
There is no suitable definition for the term AGV yet.	2.43	2	3	2.64	2.00	0.026(*)	2.22	2.60	0.094
There is no suitable definition for the term AMR yet.	3.08	3	3	3.06	3.12	0.660	3.04	3.10	0.914
There is no suitable definition for the term mobile robot yet.	3.45	4	4	3.42	3.53	0.629	3.48	3.43	0.688

(*) indicating significant results.

Across the overall sample, the first three statements yield clear evidence that the current terminology is perceived as problematic: participants agree that there are different perceptions in the communication of terms (mean overall = 4.40, median = 4, mode = 5), that definitions are conflicting (mean overall = 3.96, median = 4, mode = 4) and that uniform definitions are lacking (mean overall = 4.13, median = 4, mode = 5). The corresponding distributions underline the strength of this assessment. For Statement 1, almost all responses fall on the agreement side, with virtually no complete disagreement, see Subfigure Figure 4a. For Statement 2, agreement remains dominant, while a non-negligible share selects neutral or mild disagreement, indicating that perceived conflict is present but not unanimous, see Subfigure Figure 4b. For Statement 3, the distribution again concentrates on agreement with fewer neutral responses and limited disagreement, see Subfigure Figure 4c. Taken together, these three items may provide a empirical rationale for the present study: experts consistently report that the terminology landscape is characterized by divergent interpretations and insufficient convergence.

Looking at subgroup differences in Table 6, patterns are largely stable across R&D and management splits, with only a few notable deviations. Differences between management and non-management are small for the three motivating statements, suggesting that perceptions of inconsistency and non-uniformity are shared broadly across organizational roles. This is also reflected by the CI for the management comparison, which overlap the no-effect value of 0.5 for all statements (95% CIs: [0.209, 0.505] to [0.367, 0.686]). The R&D vs. non-R&D comparisons also show no clear differences for the first three statements, indicating that the perception of divergent and conflicting terminology is not limited to one functional subgroup.

Beyond these core items, the remaining statements refine the picture. Respondents show only moderate agreement that existing definitions are outdated, suggesting that the perceived problem is not merely one of age. Furthermore, participants differentiate between terms when judging whether suitable definitions exist: agreement that there is no suitable definition yet is lower for DTV and AGV than for AMR and MR. For DTV and AGV the R&D group reports slightly higher agreement with no suitable definition than the non-R&D group, pointing to a more critical stance among R&D respondents regarding definitional adequacy. This difference is statistically significant for both DTV (BM- p=0.047 ) and AGV (BM- p=0.026 ). The corresponding CIs are entirely above 0.5 for both statements (DTV: 95% CI [0.504, 0.809]; AGV: 95% CI [0.532, 0.837]), indicating a robust tendency toward stronger agreement in the R&D group. Overall, Question 10 indicates broad consensus that terminology is inconsistently perceived and insufficiently uniform, while also revealing that experts’ concerns differ by term, supporting the need to empirically investigate how these labels are understood and applied in practice.

Responses to Question 11 provide an overview of additional terminology that participants are familiar with, based on a predefined list of commonly used terms in the domain of robotics, see Table 7. Participants were able to select multiple entries and an open Other option allowed the inclusion of further terms not covered by the predefined list. The results show that several additional terms are widely recognized, such as Industrial Truck (n=34) , AGVS (n=32) and Driverless Truck (n=29) , indicating a broad awareness of alternative designations beyond the core terms examined in this study. At the same time, familiarity with other predefined terms (e.g., LGV, IMR, IGV) varies considerably across participants, suggesting that their usage may be context- or application-specific. The free-text responses provided under Other did not introduce a large number of entirely novel concepts but primarily revealed variants or extensions of existing terminology (e.g., autonomous AGV, DTV, AMR). Overall, these findings indicate that while a broad set of additional terms is known within the expert community, new terminology tends to emerge incrementally as variations of established concepts rather than as clearly distinct new classes.

**TABLE 7:** Results of question 11: Total counts of additional used terms.

Term	Mentions
AGVS (automated guided vehicle system)	32
AIV (automated industrial vehicle)	6
AGC (automated guided cart)	7
AVS (autonomous vehicle system)	8
LGV (laser guided vehicle)	20
IGV (intelligent guided vehicle)	11
RGV (rail guided vehicle)	7
DT (driverless truck)	29
IMR (industrial mobile robot)	14
IT (industrial truck)	34
Other	9

The purpose of the free text Question 12 was to elicit experts’ explanations for why the terms DTV, AGV, AMR and MR are perceived inconsistently and to collect suggestions for how terminology and definitions could be improved in practice. Overall, responses point to marketing-driven terminology proliferation as a primary driver: manufacturers intentionally introduce or adopt “[…] more modern-sounding” labels to differentiate products and several participants described AGV and AMR as having become “[…] marketing buzzwords”. This is closely linked to a perceived lack of uniform and enforced standards even when definitions exist, they are not consistently applied and some respondents argued that “[…] precise definitions are merely academic games that have no significant relevance in practice”. A third theme concerns technological evolution and hybrid systems, where rapid development and mixed navigation concepts blur category boundaries and make fixed labels harder to maintain. Experts emphasized application- and customer-specific usage, noting that terms are often adapted to a sector or use case rather than used as stable technical categories. In addition, linguistic and cultural factors were frequently cited, especially translation issues between German and English terminology and regional differences (e.g., AGV perceived as outdated in some contexts). Finally, participants mentioned limited awareness and education as well as skepticism toward over-terminologization, warning that continuously adding new acronyms can itself increase confusion. Taken together, the responses suggest that differing perceptions stem less from a complete absence of definitions and more from the interplay of market communication, uneven standard uptake, technological convergence, contextual usage and language-related variation.

Question 13 was used to capture additional feedback on the survey design and the broader terminology debate. Participants emphasized the importance of offering explicit non-response options to avoid forcing arbitrary selections and potentially biasing results. To ensure analytical clarity, we did not include empty submissions or no answer entries in the qualitative summary. This exclusion was applied deliberately because non-responses do not provide interpretable content about perceptions or suggested improvements retaining them would inflate the apparent volume of feedback without adding information. Beyond survey-design feedback, the comments reinforce the paper’s central theme that terminology is often shaped by non-technical drivers.

### 4.1 Ambiguities between normative terminology and expert perceptions

While standards, regulations, interoperability initiatives, and academic publications provide terminology for industrial mobile robotics, they do not necessarily determine how labels are understood and used in professional communication. Motivated from the results of Question 10, our survey results show ambiguities between what is formally defined or implicitly framed in normative references and the perceptions that experts have when describing, distinguishing and attributing properties to DTV, AGV, AMR and MR. In particular, broad vocabulary definitions (e.g., for MR), safety-scope framings (e.g., acknowledging multiple coexisting labels), and interface-centered initiatives (e.g., focusing on implementable message sets) may still leave room for interpretation. As a consequence, experts rely on recurring capability cues to stabilize meaning in practice, even when such cues are not explicitly part of the standard definition. Table 8 summarizes the observed mismatches between normative terminology coverage and the survey evidence across Question 5 to Question 13.

**TABLE 8:** Ambiguities between standards and references from Table 1 to perceptions of experts.

Reference, name and landscape	Term coverage	Deviations in expert perceptions (Q5–Q13)
ISO8373 International Organization for Standardization, (2021) Standard (vocabulary)	MR: D; AGV: M; AMR: —; DTV: —	MR is enriched beyond “travels under its own control”: Q5, Q8. MR is treated as an broad term conflicting with implicit requirements: Q7. Boundary ambiguity persists: Q6
ISO19649:2017 International Organization for Standardization, (2017) Standard (vocabulary)	MR: M/D; AGV: —; AMR: —; DTV: —	Vocabulary-level MR does not resolve professional granularity: Q5, Q7. Need for qualifiers: Q10
ISO3691-4 International Organization for Standardization, (2023) Standard (safety)	MR: —; AGV: M; AMR: M; DTV: —	Practical distinctions exceed scope framing: Q6, Q8. Every robot has to be safe: Q5, Q8
ISO5053-1 International Organization for Standardization, (2020) Standard (vocabulary)	MR: —; AGV: —; AMR: —; DTV: —	Truck vocabulary does not match the rest of the terms.: Q11, Q12, Q13. Application-specific expectations dominate: Q5
VDI2510 Verein Deutscher Ingenieure, (2005) guideline (domain practice)	MR: —; AGV: D; AMR: —; DTV: D (in German)	AGV–DTV treated as interchangeable: Q6, Q8. Navigation connotation is stronger than the definition alone: Q5
DTV usage examples Hansen and Gillert (2008), Arvato (2020), BMW Group (2024), Ehrhardt Partner Group, (2026) practice (industry)	MR: —; AGV: —; AMR: —; DTV: M/D	Label coexistence (AGV/DTV) does not prevent conflation: Q6. DTV functions as a not exact AGV-alias in experts’ minds: Q6, Q8. DTV triggers translation issues: Q5, Q12
ISO/DIS21423 International Organization for Standardization (2026c) Standard (draft)	MR: —; AGV: —; AMR: M; DTV: —	AMR meaning is inferred from capabilities, not from a stable definition: Q5, Q8. Non-uniformity remains despite AMR prominence: Q10
ANSI/RIA R15.08-1-2020 Institute (2020) Standard (safety, US)	MR: —; AGV: D; AMR: D; DTV: —	Definition-aligned distinction is present but not universal: Q6, Q8, Q10. Boundary reliance on navigation/autonomy cues: Q5
Regulation (EU) 2023/1230 European Parliament and Council of the European Union, (2023) Regulation (safety, EU)	MR: —; AGV: —; AMR: M; DTV: —	Regulatory safety expectations map to AMR capability cues: Q5, Q8. Terminology remains unstable: Q10
VDA5050 VDA 5050 Community (2026) industry initiative from Germany (interoperability)	MR: M; AGV: M; AMR: —; DTV: —	Shift toward MR does not remove ambiguity: Q5, Q7. Interoperability semantics may override vocabulary boundaries: Q8, Q10
MassRobotics AMR interoperability Standard MassRobotics (2021) industry initiative from US (interoperability)	MR: —; AGV: —; AMR: M; DTV: —	AMR is treated as the modern/autonomous class: Q5, Q8. Ecosystem-level focus does not ensure global convergence: Q11, Q10
Academic working definitions (examples) Lackner et al. (2024), Fragapane et al. (2021), Zhang et al. (2023) academic literature	MR: M/D; AGV: M/D; AMR: M/D; DTV: —	Capabilities appear in experts’ perceptions: Q5, Q8. Perceived inconsistency persists: Q10

The ambiguities mapped in Table 8 lead to three implications relevant to the design, communication and use of terminology in industrial mobile robotics which are discussed in the following.

First, terms should be communicated together with a minimal set of technical properties or dimensions that match how experts form expectations. Those can be inspired from Question 5 because if they are not specified, the experts will interpret them anyway since they interact with the real systems. Therefore, when terms are used in publications, procurement specifications, safety documentation or system descriptions, we recommend pairing the label with at least these qualifiers. Our proposal is therefore not to create new definitions, but rather to harmonize existing ones and, where necessary, to refine them or communicate them more effectively. This can help to reduce the expectation gap by making explicit the cues that experts already rely on in practice.

Second, broad labels such as MR should be treated as category headers rather than as stand-alone descriptors. Although MR is formally defined at the vocabulary level as a robot that can travel under its own control, experts commonly attribute broader properties to MR, including autonomy and sensing and simultaneously express mixed views about whether the term is sufficiently precise for professional use (see Question 5 or Question 7). Future technologies, as discussed in Question 9, may intensify the debate on the broad label of MRs. In addition, MR was perceived as only weakly separable from AMR (Question 6), indicating that MR without qualifiers can obscure conceptual boundaries. We therefore suggest using MR as a top-level category in communication (e.g., “MR systems in industrial applications”) and specifying the intended subcategory (AGV, AMR, DTV) or capability profile when the aim is to convey operational requirements.

Third, terminology governance should acknowledge translation-driven and ecosystem-driven label dynamics rather than assuming purely top-down standard adoption. In an ideal world, the translation of AGV should therefore correspond exactly to DTV in the survey. The survey shows that the correspondences are high but not identical. The spoken language and the community can give rise to different expectations. Respondents report that definitions are perceived as non-uniform and partially conflicting (Question 10) and explicitly attribute this to marketing-driven naming, hybrid technical developments and regional or language-related usage conventions (Question 12). While a definition of AMR is not yet perceived it is associated with modernity, decentralized control and advanced autonomy cues (Question 5, Question 8). This implies that standardization efforts aiming at terminology alignment should complement vocabulary definitions with practical mapping guidance, e.g., explicit synonym/alias handling (AGV–DTV) and usage examples that reflect real deployment contexts. Similarly, interoperability initiatives (e.g., VDA5050) should document term usage decisions transparently, as their working groups effectively shaping labels through message semantics and thereby shape community meaning.

## 5 Conclusion and implications for future work

With this work, we investigated how experts in industrial mobile robotics perceive and use the terms driverless transport vehicle (DTV), automated guided vehicle (AGV), autonomous mobile robot (AMR) and mobile robot (MR). Motivated by the role of terminology as a cue for expectations in human–robot interaction (HRI), we conducted a targeted expert survey. Across the survey questions, the results suggest that terminology is not merely a neutral labeling choice. Instead, the four investigated terms evoke different perceptions along recurring dimensions.

Findings include that experts perceived AGV and DTV as largely non-distinguishable (Question 6 RE = −0.51 rescaled on a scale from 1.0 to −1.0), indicating that DTV is widely treated as a synonym or translation variant of AGV in practice. Second, AGV and AMR were perceived as clearly more distinguishable (Question 6 RE = 0.37), confirming that experts associate these labels with meaningfully different capability profiles. Third, respondents reported broad agreement that the current terminology landscape is inconsistent, including different perceptions in communication (Question 10 Statement 1) and a lack of uniform definitions (Question 10 Statement 3).

By linking these findings to the terminology landscape in standards, regulations, interoperability initiatives, and academic working definitions, the study highlights a gap between normative terminology and perceived terminology. Vocabulary standards provide broad definitions (especially for MR), safety documents often frame scopes rather than sharp category boundaries, and interoperability initiatives prioritize implementable semantics and may evolve label usage over time. In parallel, academic literature frequently introduces pragmatic working definitions tailored to specific research perspectives. Our results indicate that experts actively fill such definitional gaps using capability cues and contextual knowledge, which can stabilize meaning locally but also creates ambiguity and expectation gaps across stakeholder groups. Beyond documenting expert perceptions, the results support actionable implications for the design, communication, and use of terminology in industrial mobile robotics.

Based on these findings, we propose three practical implications derived from a German-speaking expert sample for addressing terminology-related ambiguities and their consequences. First, terminology should be simplified rather than expanded with additional labels. Since DTV was perceived as largely overlapping with AGV, the use of AGV as the primary term appears sufficient in most international and technical contexts, while DTV can be treated as a German translation-related variant rather than a separate category. The three most relevant labels, AGV, AMR, and MR, should then be distinguished through explicit contextual qualifiers, such as navigation concept, level of autonomy, application context, interaction requirements, and relevant safety assumptions. This approach does not introduce another terminology layer, but clarifies how existing labels are used in a given context and reduces the risk of turning terminology into marketing buzzwords or academic wordplay. It also reduces expectation gaps by making explicit which capabilities are actually meant when a term is used. Second, broad terms such as MR should be treated as umbrella categories rather than precise system descriptions. When MR is used in publications, procurement documents, standards, or training material, the intended subcategory or capability profile should be specified to avoid unclear assumptions about autonomy, sensing, or operational behavior. Third, standardization bodies, interoperability initiatives, and industrial working groups should explicitly document synonym relations, translation issues, and changes in terminology over time. For example, the close relation between AGV and DTV should be communicated transparently, while distinctions between AGV and AMR should be linked to observable technical and operational criteria. Such guidance would not eliminate all terminology differences, but it would make their consequences more manageable in HRI-relevant contexts such as planning, safety assessment, user training, and stakeholder communication.

This study has several limitations. First, the sample consists of 53 complete responses from a targeted expert recruitment strategy and most respondents were based in Germany. Thus, the results may not generalize to other regions or linguistic communities where term usage conventions differ. Incomplete responses were not saved, and therefore there is no possibility for further comparison. Accordingly, only the sample of 53 experts could be analyzed here. A larger, internationally designed study should be conducted in this regard. Also, the survey captures self-reported perceptions rather than observed behavior in real-world environments, procurement processes, or deployment projects. A further limitation concerns the subjective nature of Likert-type response categories: although respondents selected ordered categories, the perceived distance between adjacent response options may differ between individuals and between scale positions, so mean values, and linear transformations are used only as exploratory descriptive approximations and should not be interpreted as exact interval-scale measurements.

Future work should therefore extend the empirical basis of terminology perception in at least four directions. The results of this work enable the creation of harmonized definitions that combine the views of experts and those found in the literature. To this end, the state of the art must be recorded alongside the standards by means of a systematic literature analysis. It is essential to continue this research in light of new technological developments in the coming years, as many of these advancements are increasing the diversity in mobile robotics. Emerging technologies, such as humanoid and quadruped robots, are likely to become increasingly common in industrial settings. However, they are not yet widely established in practice, so a study such as the one presented here is not yet feasible. Also, replication with larger and more internationally diverse samples is needed to assess cultural and regional differences. We encourage others to adapt our contribution for use in other communities in other countries and provide a replicable approach for doing so. Similarly, future studies should expand the questionnaire to include safety, trust, and systems engineering perspectives. This would make it possible to investigate how terminology-related expectation gaps affect not only perceived robot capabilities, but also safety assumptions, trust calibration, responsibility allocation, and the understanding of the robotic system as part of a broader sociotechnical system. Finally, communicating the harmonized definitions to the standardization initiatives and working groups can create a uniform understanding within the community and, ultimately, be incorporated into normative standards. This would contribute to overcoming the challenge posed by the various labels in HRI research.

In conclusion, our survey demonstrates that terminology in industrial mobile robotics carries expectation cues and that experts’ mental models diverge in ways that are not fully resolved by existing standards or academic definitions. By mapping normative terminology coverage to empirical perceptions, we provide evidence for where communication-relevant gaps persist and offer guidance for using terminology in ways that reduce misunderstandings across stakeholders.

## Data Availability

The datasets presented in this study can be found in online repositories. The names of the repository/repositories and accession number(s) can be found below: https://zenodo.org/records/18782395?token&equals;eyJhbGciOiJIUzUxMiJ9.eyJpZCI6IjJhNWJmZjIyLTM3YmQtNGEwNC05M2U0LWE1NTkzNmVhNjkyOCIsImRhdGEiOnt9LCJyYW5kb20iOiI0MTk5OWYyNDY0YWZmZjE4OTZlYTMzM2Y0MmQwMjg4MyJ9.7Eo4k5uEvtyB84pP0LJTp3s4GDHDayjxRxLmpqv99FBVlYJBObqdooESk3EMegKeP4SDS65P3OP9Ue34yClVuw.
