# Supplementary material for: Ambiguous definitions of industrial mobile robots: a survey of German-speaking experts
Source: Front Robot AI. 2026 Aug 28;13:1837981. doi: 10.3389/frobt.2026.1837981 (PMC13561907; doi:10.3389/frobt.2026.1837981)
Supplement: Supplementary file 1 [file DataSheet1.pdf]

Supplementary Material: Survey Perception of the Terms AGV, AMR, DTV and MR

INTRODUCTION TEXT FOR PARTICIPANTS

Thank you for taking the time to participate in this survey!

In the world of robotics, terms such as AGV (Automated Guided Vehicle), AMR (Autonomous Mobile Robot), DTV (Driverless Transport Vehicle) or MR (Mobile Robot) are frequently used – but not always uniformly. The aim of the survey is to gain a better understanding of how these terms are used and distinguished from one another in everyday working life. This survey is not a test of technical knowledge, but rather a survey of your personal perception and understanding of the terms. There are no right or wrong answers in the survey. All data will be collected anonymously. It will not be possible to identify individuals directly or indirectly. The data will be evaluated exclusively for the purpose of the survey. If you have any questions or comments, please contact the corresponding author. There are 13 questions in this survey.

Q1: POSITION IN THE COMPANY

Question: What is your position in the company you work for?

Type: Single choice (with free-text option)

- Chief executive management / Executive Board / Owner
- Executive management / Division management
- Department management / Team management / Group management
- Skilled worker / Specialist / Employee without management responsibility
- Assistance / Secretariat
- Trainees / Interns / Working students
- Other: _______________

Q2: FUNCTIONAL AREA

Question: In which functional area do you work in your company?

Type: Single choice

- Purchasing / Procurement
- Service creation / Production
- Distribution / Sales
- Finance / Controlling / Accounting
- Human resources
- Customer service / Support
- Marketing / Communication
- Research / Development

Q3: AGE

Question: Please enter your age.

Type: Open numeric input between 18 and 99

Q4: COUNTRY

Question: Please specify the country in which you work.

Type: Single choice with list of all countries

Q5: SPONTANEOUS ASSOCIATIONS WITH ROBOT TERMINOLOGY

Question: Which three terms come to your mind when you think of Automated Guided Vehicles (AGV), Autonomous Mobile Robots (AMR), Driverless Transport Vehicles (DTV) and Mobile Robots (MR)? Use one text box per term. Please answer as spontaneously as possible without thinking too long.

Type: Open text (three entries per item)

- Automated Guided Vehicle (AGV)
- Autonomous Mobile Robot (AMR)
- Driverless Transport Vehicle (DTV)
- Mobile Robot (MR)

Q6: DISTINGUISHABILITY OF TERMS

Question: Are the following pairs of terms distinguishable/separable for you? Please select the appropriate answer for each line from 1 (terms are not distinguishable/separable at all) to 5 (terms are clearly distinguishable/separable)

Type: Likert scale (1–5) *1 = Not distinguishable at all, 5 = Clearly distinguishable*

- AGV and AMR
- AGV and DTV
- AMR and DTV
- AMR and Mobile Robot
- AGV and Mobile Robot
- DTV and Mobile Robot

Q7: GENERAL STATEMENTS ON TERMINOLOGY

Question: Please select the answer that you think is most appropriate for the following statements.

Type: Likert scale (1–5) *1 = Disagree completely, 5 = Agree completely*

- The terms (DTV, AGV and AMR) are subcategories of Mobile Robots.
- The term Mobile Robot is too vague for professional use.
- The term Mobile Robot is sufficient for professional use.

- For professional use, further abstraction can be applied and terms such as robot or carriage can be used.
- The terms depend heavily on the application.
- The distinction is not relevant to my work.
- The term AGVS (Automated Guided Vehicle System) can be used to designate individual vehicles.

Q8: ASSIGNMENT OF TECHNICAL CHARACTERISTICS

Question: Match the following characteristics with the terms. Tick the box if the term fits the statement. Multiple selections are possible.

Type: Multiple choice matrix (checkboxes) **Terms:** AGV, AMR, DTV, Mobile Robot

- Drives on fixed routes (track-bound)
- Can plan routes in real time
- Moves exclusively on the ground
- Recognizes and avoids obstacles on its own
- Features comprehensive sensor technology (beyond basic safety)
- Can collaborate with people
- Controlled by a central instance
- Controlled decentralized
- Used in logistics
- Used in production
- Used in public spaces
- Used in private spaces

Q9: CLASSIFICATION OF RELATED TECHNOLOGIES

Question: How would you classify the following technologies?

Type: Multiple choice matrix (single selection per row)

- Cobots (Collaborative Robots)
- Drones / UAVs (Unmanned Aerial Vehicles)
- Humanoid Robots

Options:

- Part of Mobile Robots
- Part of AGVs
- Part of DTVs
- Part of AMRs
- Own class
- I don’t know

Q10: PERCEPTION OF DEFINITIONS AND STANDARDS

Question: Please select the answer that you think is most appropriate for the following statements.

Type: Likert scale (1–5) *1 = Disagree completely, 5 = Agree completely*

- In the communication of terms (DTV, AGV, AMR, Mobile Robot), there are different perceptions.
- There are conflicting definitions of the terms (DTV, AGV, AMR, Mobile Robot).
- There is a lack of uniform definitions of the terms.
- Existing definitions are outdated.
- There is no suitable definition for the term DTV yet.
- There is no suitable definition for the term AGV yet.
- There is no suitable definition for the term AMR yet.
- There is no suitable definition for the term Mobile Robot yet.

Q11: FAMILIARITY WITH ADDITIONAL TERMS

Question: Which of these additional terms have you already heard or used yourself?

Type: Multiple choice (checkboxes)

- AGVS (Automated Guided Vehicle System)
- AIV (Automated Industrial Vehicle)
- AGC (Automated Guided Cart)
- AVS (Autonomous Vehicle System)
- LGV (Laser Guided Vehicle)
- IGV (Intelligent Guided Vehicle)
- RGV (Rail Guided Vehicle)
- DT (Driverless Truck)
- IMR (Industrial Mobile Robot)
- IT (Industrial Truck)
- Other: _______________

Q12: OPEN FEEDBACK TO DIFFERENT DEFINITIONS

Question: If you feel that the terms are perceived too differently or that there are insufficient definitions, what do you think is the reason for this and what could be changed?

Type: Open text

Q13: ADDITIONAL COMMENTS

Question: Do you have any further comments?

Type: Open text
